# Erratum to: Fanconi-Bickel Syndrome - Mutation in *SLC2A2* Gene

**DOI:** 10.1007/s12098-016-2197-9

**Published:** 2016-08-01

**Authors:** Mohit Kehar, Sunita Bijarnia, Sian Ellard, Jayne Houghton, Renu Saxena, I. C. Verma, Nishant Wadhwa

**Affiliations:** 1 Division of Pediatric Gastroenterology and Hepatology, Sir Ganga Ram Hospital, Rajendra Nagar, New Delhi, India; 2 Center of Medical Genetics, Sir Ganga Ram Hospital, Rajendra Nagar, New Delhi, 110060 India; 3 Department of Molecular Genetics, Foundation Trust, Royal Devon and Exeter NHS, Exeter, EX2 5AD UK; 4 Institute of Biomedical and Clinical Science, Medical School, University of Exeter, Exeter, EX2 5DW UK

**Erratum to: Indian J Pediatr 81(11):1237–1239**

**DOI 10.1007/s12098–014–1487-3**

The original published online version's copyright line has to be corrected as Open Access (see updated copyright line shown above). Open access statement is also included below.

